# The airborne lifetime of small speech droplets and their potential importance in SARS-CoV-2 transmission

**DOI:** 10.1073/pnas.2006874117

**Published:** 2020-05-13

**Authors:** Valentyn Stadnytskyi, Christina E. Bax, Adriaan Bax, Philip Anfinrud

**Affiliations:** ^a^Laboratory of Chemical Physics, National Institute of Diabetes and Digestive and Kidney Diseases, National Institutes of Health, Bethesda, MD 20892-0520;; ^b^Perelman School of Medicine, University of Pennsylvania, Philadelphia, PA 19104

**Keywords:** COVID-19, speech droplet, independent action hypothesis, respiratory disease, disease transmission

## Abstract

Speech droplets generated by asymptomatic carriers of severe acute respiratory syndrome coronavirus 2 (SARS-CoV-2) are increasingly considered to be a likely mode of disease transmission. Highly sensitive laser light scattering observations have revealed that loud speech can emit thousands of oral fluid droplets per second. In a closed, stagnant air environment, they disappear from the window of view with time constants in the range of 8 to 14 min, which corresponds to droplet nuclei of *ca.* 4 μm diameter, or 12- to 21-μm droplets prior to dehydration. These observations confirm that there is a substantial probability that normal speaking causes airborne virus transmission in confined environments.

It has long been recognized that respiratory viruses can be transmitted via droplets that are generated by coughing or sneezing. It is less widely known that normal speaking also produces thousands of oral fluid droplets with a broad size distribution (*ca.* 1 μm to 500 μm) (1, 2). Droplets can harbor a variety of respiratory pathogens, including measles (3) and influenza virus (4) as well as *Mycobacterium tuberculosis* (5). High viral loads of severe acute respiratory syndrome coronavirus 2 (SARS-CoV-2) have been detected in oral fluids of coronavirus disease 2019 (COVID-19)−positive patients (6), including asymptomatic ones (7). However, the possible role of small speech droplet nuclei with diameters of less than 30 μm, which potentially could remain airborne for extended periods of time (1, 2, 8, 9), has not been widely appreciated.

In a recent report (10), we used an intense sheet of laser light to visualize bursts of speech droplets produced during repeated spoken phrases. This method revealed average droplet emission rates of *ca.* 1,000 s^−1^ with peak emission rates as high as 10,000 s^−1^, with a total integrated volume far higher than in previous reports (1, 2, 8, 9). The high sensitivity of the light scattering method in observing medium-sized (10 μm to 100 μm) droplets, a fraction of which remain airborne for at least 30 s, likely accounts for the large increase in the number of observed droplets. Here, we derive quantitative estimates for both the number and size of the droplets that remain airborne. Larger droplets, which are also abundant but associated with close-proximity direct virus transfer or fomite transmission (11), or which can become resuspended in air at a later point in time (12), are not considered here.

According to Stokes’ law, the terminal velocity of a falling droplet scales as the square of its diameter. Once airborne, speech-generated droplets rapidly dehydrate due to evaporation, thereby decreasing in size (13) and slowing their fall. The probability that a droplet contains one or more virions scales with its initial hydrated volume, that is, as the cube of its diameter, *d*. Therefore, the probability that speech droplets pass on an infection when emitted by a virus carrier must take into account how long droplet nuclei remain airborne (proportional to *d*^−2^) and the probability that droplets encapsulate at least one virion (proportional to *d*^3^), the product of which is proportional to *d*.

The amount by which a droplet shrinks upon dehydration depends on the fraction of nonvolatile matter in the oral fluid, which includes electrolytes, sugars, enzymes, DNA, and remnants of dehydrated epithelial and white blood cells. Whereas pure saliva contains 99.5% water when exiting the salivary glands, the weight fraction of nonvolatile matter in oral fluid falls in the 1 to 5% range. Presumably, this wide range results from differential degrees of dehydration of the oral cavity during normal breathing and speaking and from decreased salivary gland activity with age. Given a nonvolatile weight fraction in the 1 to 5% range and an assumed density of 1.3 g⋅mL^−1^ for that fraction, dehydration causes the diameter of an emitted droplet to shrink to about 20 to 34% of its original size, thereby slowing down the speed at which it falls (1, 13). For example, if a droplet with an initial diameter of 50 μm shrinks to 10 μm, the speed at which it falls decreases from 6.8 cm⋅s^−1^ to about 0.35 cm⋅s^−1^. The distance over which droplets travel laterally from the speaker’s mouth during their downward trajectory is dominated by the total volume and flow velocity of exhaled air (8). The flow velocity varies with phonation (14), while the total volume and droplet count increase with loudness (9). Therefore, in an environment of stagnant air, droplet nuclei generated by speaking will persist as a slowly descending cloud emanating from the speaker’s mouth, with the rate of descent determined by the diameter of the dehydrated speech droplet nuclei.

The independent action hypothesis (IAH) states that each virion has an equal, nonzero probability of causing an infection. Validity of IAH was demonstrated for infection of insect larvae by baculovirus (15), and of plants by Tobacco etch virus variants that carried green fluorescent protein markers (16). IAH applies to systems where the host is highly susceptible, but the extent to which IAH is valid for humans and SARS-CoV-2 has not yet been firmly established. For COVID-19, with an oral fluid average virus RNA load of 7 × 10^6^ copies per milliliter (maximum of 2.35 × 10^9^ copies per milliliter) (7), the probability that a 50-μm-diameter droplet, prior to dehydration, contains at least one virion is ∼37%. For a 10-μm droplet, this probability drops to 0.37%, and the probability that it contains more than one virion, if generated from a homogeneous distribution of oral fluid, is negligible. Therefore, airborne droplets pose a significant risk only if IAH applies to human virus transmission. Considering that frequent person-to-person transmission has been reported in community and health care settings, it appears likely that IAH applies to COVID-19 and other highly contagious airborne respiratory diseases, such as influenza and measles.

## Results and Discussion

The output from a green (532 nm) Coherent Verdi laser operating at 4-W optical power was transformed with spherical and cylindrical optics into a light sheet that is ∼1 mm thick and 150 mm tall. This light sheet passed through slits centered on opposite sides of a cubic 226-L enclosure. When activated, a 40-mm, 12-V muffin fan inside the enclosure spatially homogenizes the distribution of particles in the enclosure. A movie showing the arrangement is available (17). Movie clips of speech droplet nuclei were recorded at a frame rate of 24 Hz with high-definition resolution (1,920 × 1,080 pixels). The camera lens provided a horizontal field of view of ∼20 cm. Therefore, the volume intercepted by the light sheet and viewed by the camera is ∼30 cm^3^. The total number of particles in the enclosure can be approximated by multiplying the average number of particles detected in a single movie frame by the volume ratio of the enclosure to the visualized sheet, which is ∼7,300. Slow convection currents, at speeds of a few centimeters per second, remained for the duration of the recording. These convection currents are attributed to a 0.5 °C temperature gradient in the enclosure (bottom to top) that presumably is due to heat dissipated by the iPhone11 camera, which was attached to the front side of the enclosure. Since the net air flux across any horizontal plane of the enclosure is zero, this convection does not impact the average rate at which droplet nuclei fall to the bottom of the enclosure.

**Fig. 1.**
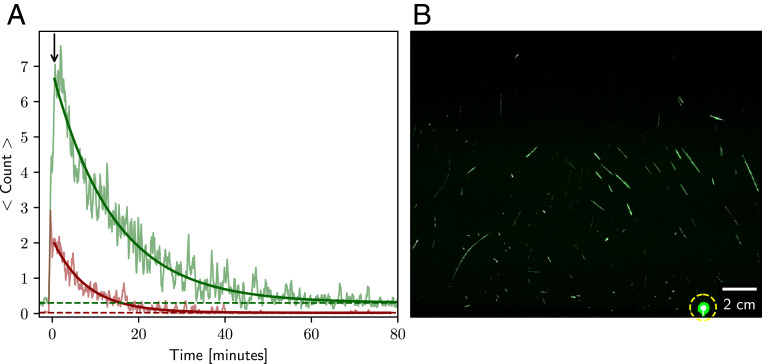
Light scattering observation of airborne speech droplet nuclei, generated by a 25-s burst of repeatedly speaking the phrase “stay healthy” in a loud voice (maximum 85 dB_B_ at a distance of 30 cm; average 59 dB_B_). (*A*) Chart of particle count per frame versus time (smoothed with a 24-s moving average), with the red curve representing the top 25% in scattering brightness and the green curve representing the rest. The bright fraction (red) decays with a time constant of 8 min, and the dimmer fraction (green) decays with a time constant of 14 min. Both exponential decay curves return to their respective background level of *ca.* 0 (red horizontal dashed line) and 0.4 (green dashed line) counts per frame. Time “0” corresponds to the time the stirring fan was turned off. The 25-s burst of speaking started 36 s before time 0. The black arrow (at 0.5 min) marks the start of the exponential fits. (*B*) Image of the sum of 144 consecutive frames (spanning 6 s) extracted shortly after the end of the 25-s burst of speaking. The dashed circle marks the needle tip used for focusing the camera. The full movie recording is available in ref. 17, with time “0” in the graph at time point 3:38 in the movie.

With the internal circulation fan turned on, the enclosure was purged with HEPA-filtered air for several minutes. Then, the purge shutter was closed, the movie clip was started, the speaker port was opened, and the enclosure was “filled” with speech droplets by someone repeating the phrase “stay healthy” for 25 s. This phrase was chosen because the “th” phonation in the word “healthy” was found to be an efficient generator of oral fluid speech droplets. The internal fan was turned off 10 s after speech was terminated, and the camera continued recording for 80 min. The movie clip was analyzed frame by frame to determine the number of spots/streaks whose maximum single-pixel intensity exceeded a threshold value of 30. Fig. 1 charts the time-dependent decrease in the number of scattering particles detected. We are not yet able to quantitatively link the observed scattered light intensity to the size of the scattering particle because the light intensity varies across the sheet. However, the brightest 25% were found to decay more quickly than the dimmer fraction, with the two curves reasonably well described by exponential decay times of 8 and 14 min, respectively (Fig. 1*A*). These fits indicate that, near time 0, there were, on average, approximately nine droplet nuclei in the 30-cm^3^ observation window, with the larger and brighter nuclei (on average) falling to the bottom of the enclosure at faster speeds than the smaller and dimmer ones.

With the assumption that the contents of the box are homogenized by the muffin fan at time 0, the average number of droplets found in a single frame near time 0 corresponds to *ca.* 66,000 small droplets emitted into the 226-L enclosure, or *ca.* 2,600 small droplet nuclei per second of speaking. If the particle size distribution were a delta function and the particles were uniformly distributed in the enclosure, the particle count would be expected to remain constant until particles from the top of the enclosure descend to the top of the light sheet, after which the particle count would decay linearly to background level. The observation that the decay profiles are approximately exponential points to a substantial heterogeneity in particle sizes, even after binning them into two separate groups.

The weighted average decay rate (0.085 min^−1^) of the bright and dim fractions of particles (Fig. 1*A*) translates into a half-life in the enclosure of *ca.* 8 min. Assuming this half-life corresponds to the time required for a particle to fall 30 cm (half the height of the box), its terminal velocity is only 0.06 cm⋅s^−1^, which corresponds to a droplet nucleus diameter of ∼4 μm. At the relative humidity (27%) and temperature (23 °C) of our experiment, we expect the droplets to dehydrate within a few seconds. A dehydrated particle of 4 μm corresponds to a hydrated droplet of *ca.* 12- to 21-μm diameter, or a total hydrated volume of ∼60 nL to 320 nL for 25 s of loud speaking. At an average viral load of 7 × 10^6^ per milliliter (7), we estimate that 1 min of loud speaking generates at least 1,000 virion-containing droplet nuclei that remain airborne for more than 8 min. These therefore could be inhaled by others and, according to IAH, trigger a new SARS-CoV-2 infection.

The longest decay constant observed by us corresponds to droplets with a hydrated diameter of ≥12 μm when exiting the mouth. The existence of even smaller droplets has been established by aerodynamic particle sizer (APS) measurements (2). APS is widely used for detecting aerosol particulates and is best suited for particles in the 0.5- to 5-μm range. Morawska et al. (2) detected as many as 330 particles per second in the 0.8- to 5.5-μm range upon sustained “aah” vocalization. Considering the short travel time (0.7 s) between exiting the mouth and the APS detector, and the high relative humidity (59%) used in that study, droplet dehydration may have been incomplete. If it were 75% dehydrated at the detector, an observed 5.5-μm particle would have started as an 8.7-μm droplet when exiting the mouth, well outside the 12- to 21-μm range observed above by light scattering. This result suggests that APS and light scattering measurements form a perfect complement. However, we also note that, even while the smallest droplet nuclei effectively remain airborne indefinitely and have half-lives that are dominated by the ventilation rate, at a saliva viral load of 7 × 10^6^ copies per milliliter, the probability that a 1-μm droplet nucleus (scaled back to its originally hydrated 3-μm size) contains a virion is only 0.01%.

Our current setup does not detect every small particle in each frame of the movie, and our reported values are therefore conservative lower limit estimates. We also note that the saliva viral load shows large patient-to-patient variation. Some patients have viral titers that exceed the average titer of Wölfel et al by more than two orders of magnitude (7, 18), thereby increasing the number of virions in the emitted droplets to well over 100,000 per minute of speaking. The droplet nuclei observed in our present study and previously by APS (2, 9) are sufficiently small to reach the lower respiratory tract, which is associated with an increased adverse disease outcome (19, 20).

Our laser light scattering method not only provides real-time visual evidence for speech droplet emission, but also assesses their airborne lifetime. This direct visualization demonstrates how normal speech generates airborne droplets that can remain suspended for tens of minutes or longer and are eminently capable of transmitting disease in confined spaces.

### Data Availability Statement.

All raw data used for analysis are available in ref. 17.
